# Benefits and harms of social media use: A latent profile analysis of emerging adults

**DOI:** 10.1007/s12144-022-03473-5

**Published:** 2022-07-21

**Authors:** Brian TaeHyuk Keum, Yu-Wei Wang, Julia Callaway, Israel Abebe, Tiana Cruz, Seini O’Connor

**Affiliations:** 1 Department of Social Welfare, University of California Los Angeles, 337 Charles E. Young Drive, Los Angeles, CA 90095 USA; 2 University of Maryland, College Park, MD USA; 3 University of Southern Denmark, Odense, Denmark; 4 Refugees As Survivors New Zealand, Auckland, New Zealand

**Keywords:** Benefits, Harms, Latent profile analysis, Meaning in life, Social media

## Abstract

The rise in social media use among emerging adults in the United States has been well-documented, but researchers are still working on identifying how the type—not just the frequency—of use impacts psychological well-being. We identified “profiles” of social media use among young adults based on the frequency and purposes of use, and examined their associations with benefits and harms to psychosocial well-being, using data from 2828 incoming undergraduate students (*M*_age_ = 18.29 years; age range: 17 to 25 years). Using Latent Profile Analysis, we identified three unique profiles of individuals who used social media with varying levels of intensity across different purposes: Active Users (32.4%), Passive Users (25.3%), and Average Users (42.4%). Each profile was associated with varying levels of beneficial and harmful psychosocial outcomes. Compared to Average Users, (a) Active Users reported significantly better psychosocial well-being, but also more harmful outcomes; and (b) Passive Users experienced significantly lower levels of perceived social media benefits and social connectedness, while also reporting less problematic social media use and social media stress. Implications of these findings for research and practice are discussed.

Emerging adulthood—defined broadly as the period of time from the late teens through the 20s, with a particular focus on ages 18-25 (Arnett, 2000)—represents an important stage for the continuation of identity and psychological development. With the ubiquitous presence of social media in young adults’ lives (Pew Research Center, 2018), a growing body of research has documented the influence of social media on this developmental process (Mazalin & Moore, 2004; Subrahmanyam et al., 2008). Researchers have identified broad patterns of social media use—such as active, passive, and problematic use (Marino et al., 2018; Verduyn et al., 2017)—and both harms and benefits associated with using social media (e.g., Verduyn et al., 2017). Others have uncovered specific patterns of social media use among adolescents (e.g., Bányai et al., 2017; Kurek et al., 2017) to identify groups of individuals that seem to be at greater mental health risk.

However, questions remain about the patterns of social media use among emerging adults that may lead to benefits or vulnerability to harmful psychological effects. Also, it is unclear whether the mixed research findings related to patterns of social media use and psychosocial outcomes were associated with “what” people use social media for. The majority of research on social media use has focused on frequency and amount of use and negative psychosocial outcomes (e.g., loneliness, distress), with less attention paid to the purpose of use and beneficial outcomes (e.g., social connection, meaning in life). To address this gap and to examine both the quantity and type of social media use in a comprehensive way, we used a novel approach to examine social media use patterns (“profiles”) of emerging adults based on the frequency and purpose of social media use and their differential associations with psychosocial benefits and harms.

## Benefits and Patterns of Social Media Use

The Uses and Gratifications Theory on social media use (Katz et al., 1973; Ruggiero, 2000) suggest people use social media to fulfill their needs and motivation for gratification.

According to this theory, one major motivator for use and gratification is to connect with others online efficiently, selectively, and meaningfully, in order to fulfill their social needs and approval (Urista et al., 2009). There are a plethora of social media platforms that help individuals form their social capital, and their social media use is largely dictated by this process. Given the importance of social connection in the survival of human beings (Holt-Lunstad et al., 2010), social media has been noted as an important tool for social bonding and network building. For example, studies have found that online-mediated social connections promoted benefits and well-being among cybervictims (McLouglin et al., 2018) and buffered anxiety and isolation due to the COVID-19 pandemic (Stuart et al., 2021).

In fact, research has identified a range of social, developmental, and emotional benefits associated with social media use (Anderson & Jiang, 2018; Duggan et al., 2015; GLSEN et al., 2013). A recent study by the Pew Research Center on teenagers’ habits and experiences of using social media indicated that a majority felt more connected to their friends, interacted with a more diverse group of people, and felt supported when they used social media (Anderson & Jiang, 2018). Gender Minority groups (e.g., lesbians, gay, bisexual, and transgender youth) may particularly benefit from the use of social media and other online resources that provide information, support, and feelings of meaningfulness, as well as those social media outlets that may assist in identity development and civic connectedness (GLSEN et al., 2013). Additionally, researchers have noted the potential of social media platforms to provide a prompt for reminiscence and increased meaning in life and have demonstrated how deeper engagement with personal social media content can facilitate connections with others, enhance self-knowledge, and increase a sense of connection between present and past selves (Thomas & Briggs, 2016). Research with young adults in Australia identified “Facebook connectedness” to be distinct from other forms of social connectedness, and to be significantly associated with lower depression and anxiety and greater life satisfaction (Grieve et al., 2013).

On the contrary, some researchers have linked social media use to harmful and deleterious effects on mental health (e.g., Marino et al., 2018; Twenge et al., 2018). While the Uses and Gratifications Theory (Katz et al., 1973; Ruggiero, 2000) suggests that social connection may be a major motivator of social media use, those who use social media for connection may also experience social isolation and comparison, which can in turn yield harmful outcomes such as loneliness and self-negativity (Primack et al., 2017). Comprehensive reviews of the literature documented the negative associations between social media use and various measures of psychological well-being (Frost & Rickwood, 2017; Verduyn et al., 2017). Specifically, across dozens of cross-sectional and longitudinal studies, researchers identified a pattern of small to medium positive associations between higher levels of social media use and harmful psychological outcomes (i.e., anxiety, depression, and distorted body image), and negative relationships between levels of social media use and subjective well-being.

These seemingly conflicting findings may be explained by psychosocial variables associated with more passive or active forms of engagement. The association between social media use and well-being appears to vary with the *pattern* or *type* of social media use. Some studies suggested that passive patterns of using social media (such as browsing others’ profiles or scrolling through feeds) were associated more consistently with harmful outcomes, whereas active patterns of social media use (such as self-disclosing online, engaging with others, and sharing links) were more consistently related to beneficial outcomes (Frost & Rickwood, 2017; Verduyn et al., 2017). Other studies suggested that harmful outcomes were associated with more intense patterns of social media use that promote upward social comparison, envy, brooding, and more negative and emotional self-disclosures (which may be more likely when users are only “looking” at others’ lives and not interacting with them), whereas beneficial outcomes were associated with patterns of use that promote perceived social support, social capital, and social connectedness (Frost & Rickwood, 2017; for a summary, see Verduyn et al., 2017). Marino et al. (2018) further proposed that “problematic” patterns of use—characterized not only by a high frequency of social media use but also addiction-like symptoms and struggles with self-regulation—led to poor psychological outcomes. These researchers found that psychological distress was significantly higher among young adults across 23 independent samples who exhibited “problematic” patterns of Facebook use.

### Groups of Individuals with Distinct Patterns of Social Media Use

Noting the importance of differentiating between types of social media use, a growing number of studies examined psychosocial factors associated with different levels and patterns of use among individuals. For example, Wilson et al. (2010) examined whether social media use among 201 university students was predicted by their scores on the NEO Five-Factor Personality Inventory. They found that extroverted and less conscientious students reported significantly greater social media use and addictive inclinations. Another study showed that individuals with greater social comparison tendencies reported greater social media use (Tandoc et al., 2015). Additionally, one study demonstrated that negative collective self-esteem was associated with online social compensation among first-year college students—those who felt negatively about their social group used social media to connect with other group members and to feel better about themselves (Barker, 2009). As for other psychosocial factors, Caplan (2007) found that both self-reported loneliness and social anxiety among 343 undergraduate students were significantly associated with a preference for online interactions. In this study, they also found that social anxiety explained more variance in predicting preference for online interactions, which in turn significantly predicted greater problems in keeping up with their school, work, and social engagements. Overall, a wide range of personal characteristics and individual tendencies seem to differentiate individuals’ social media use patterns.

Against this backdrop, researchers have employed statistical analyses to identify different clusters of internet and social media use behaviors and examine whether certain patterns are associated with greater benefits or harms. For example, Eynon and Malmberg (2011) conducted Latent Profile Analysis on 1069 children and teenagers in the United Kingdom (ages 8-19) and found that, based on internet use, individuals clustered into peripheral, normative, and all-rounder/active participator groups. Using a latent segmentation approach, Alarcón-del-Amo et al. (2011) found four groups (introvert, novel, versatile, and expert-communicator) that differed in the frequency of social media use among 399 internet users (ages 16-74). Both studies found nuanced patterns of social media use that seem to be distinguished by the activities performed on social media and frequency of use. While these patterns are informative in understanding different themes of social media behaviors, they do not account for the purpose or motivation behind these behaviors that could further provide greater nuance in profiling social media use.

Beyond the level of use, other studies have examined contextual patterns of use or patterns in relation to psychological outcomes. For example, Bányai et al. (2017) used Latent Profile Analysis on data from 5961 adolescents in Europe regarding their social media addiction and found that about 4.5% were at risk for social media addiction, while 17.2% and 78.3% were at low- and no-risk, respectively. Using a similar methodology, Kurek et al. (2017) found four clusters of information and communication technology use among 933 adolescents: average use, elevated use (of all forms of technology), high video game-low social media use, and high social media and internet use. As expected, they found that adolescents in the elevated and high video game groups reported poorer identity such as false self-perception and lower self-image satisfaction, compared with the average use group. Kurek et al. (2017) also found significant relationships between the elevated and the high social media/internet use groups and self-reported problem behaviors (e.g., with friends), relative to the average group. Using a similar approach, Ilakkuvan et al. (2019) conducted a Latent Profile Analysis with 1062 young adults who used social media and found five classes: low users (lower use of social media compared to full sample), high users (higher use of social media compared to full sample), professional users (high use of professional social media such as Linkedin, low use of creative social media such as Vine), creative users (high use of vine and Tumblr, low use of Linkedin), and mainstream users (high use of Facebook and YouTube, and average use of other social media). Compared to high users, creative users had higher odds of using substances and lower odds of depressive symptoms, mainstream users had higher odds of using substances socially (alcohol and hookah), professional users had higher odds of using alcohol, cigarettes, and cigars, and low users had higher odds of using other drugs (e.g., cocaine and heroin). Altogether, findings in these studies suggested that certain patterns of use may be more likely to be associated with harmful outcomes.

### The Present Study

Based on our review, we aimed to fill two gaps in the literature. First, whereas most studies have examined social media use patterns based on the frequency of use of different social media platforms (e.g., Fardouly & Vartanian, 2015; Manago et al., 2015), we developed and employed more comprehensive social media use items differentiating patterns of use by *frequency* and *purpose* (e.g., staying connected with friends and family; networking; meeting new people; expressing ideas). Building on the Uses and Gratifications Theory (Katz et al., 1973; Ruggiero, 2000), this approach allows for a reflection of the driving forces behind certain social media activities and provides context for explaining the profiles that emerge from our study. Secondly, while most studies have focused on the psychological *harms* in relation to patterns of social media use, we also tested different social media patterns in relation to psychological *benefits*. As reviewed above and based on the Uses and Gratifications Theory that suggests motivation for social media use to fulfill social needs (Katz et al., 1973; Ruggiero, 2000), social media use presents a plethora of potential benefits in terms of social connectedness, online support, life satisfaction, and the development of one’s identity and sense of self. Therefore, we believe it is equally important to understand, promote, and capitalize on these beneficial outcomes.

We conducted Latent Profile Analysis (LPA) to uncover different types of social media users among emerging adults based on items regarding purposes of social media use. We then examined whether there may be significant differences among the groups regarding the benefits and *harms* associated with social media use. Beneficial outcomes include factors such as perceived social media-related benefits, satisfaction with life, social connectedness, and a sense of meaning in life. Harmful outcomes include factors that indicate poorer psychological well-being, such as social media-related stress, problematic social media use, and lack of social comfort on social media. Our hypotheses on these associations were contingent on the different profiles that emerged from our sample. However, based on previous research on adolescents and adults, we hypothesized that there would be higher levels of both harmful and beneficial outcomes associated with more active users, while the reverse may be true for passive users.

## Method

### Participants and Procedure

The study received Institutional Review Board approval (#316599-15). Data for the current study was drawn from archival data consisting of 2828 undergraduates (mean age = 18.3 years; age range: 17 to 25 years) who were newly entering a large Mid-Atlantic public university. The data were collected as part of an incoming student survey administered in August of 2018. We arrived at the sample size of 2828 from the original 4321 by removing cases who failed both attention-check questions (one appearing halfway and one at the end of the survey), indicated that they do not use social media, reported ages older than 25, and were missing data on the primary study variables. Because the LPA analysis strategy requires large sample sizes (Nylund-Gibson & Choi, 2018), we maximized the sample size possible.

Participants identified racially/ethnically as White (55.3%), Asian/Asian American (21.8%), Black/African American (6.9%), Chicano/Hispanic/Latino/a (4.2%), Middle Eastern/North African (1.6%), and Native Hawaiian/Pacific Islander (0.1%). One in 10 (10%) identified as bi- or multi-racial, selecting more than one of these racial backgrounds. Over half identified as female (53.1%), and 46% as male, while 0.9% identified as transgender, gender non-conforming, or having a different gender identity.

### Measures

#### Social Media Use

**Fig. 1:**
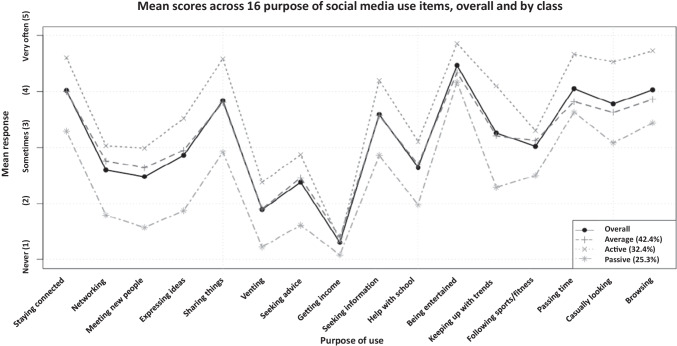
Plot of mean responses to 16 Purpose of Use variables, overall and by class

We developed items to capture students’ frequency and type of social media use. First, we asked students to rank-order social media platforms (Facebook, Instagram, Snapchat, Tumblr, Twitter, and Other—please specify) based on how much they used these social media platforms. Second, we asked students to identify their frequency of use of social media across all of these platforms, on a 1 (*never*) to 5 (*very often*) scale, for each of the 16 different purposes represented by the following categories: social connection (e.g., “staying connected with friends and family”), support and help (e.g., “seeking advice, help, or support from others”), tangible benefits (e.g., “getting income”), entertainment (e.g., following sports/fitness), and casual use (e.g., “passing time without a particular purpose;” see Fig. 1 for all 16 purposes). Third, we asked students on a scale of 1 (*never*) to 10 (*all the time*) how often they: a) “Check your social media account(s)”, b) “Actively use your social media account(s) (e.g., posting status updates, sharing links, reacting and commenting on friends’ walls, or sending messages),” and c) “Passively use your social media account(s) (e.g., scrolling through your newsfeed, looking at friends’ pages, pictures, and status updates).” We then (a) examined the total number of distinct social media platforms they engaged with and the order of the platforms based on the frequency of use, (b) calculated the average frequency for each of the 16 purposes of social media use items, and (c) calculated a mean score for their overall, active, and passive social media use.

#### Benefits of Social Media Use

##### One-Item Social Media Benefits

We developed a stand-alone item asking students to rate their agreement to the statement “I get the benefit, support, and help I need from social media.” Students indicated their response on a six-point Likert-type scale (1 = *“strongly disagree”* to 6 = *“strongly agree”*).

##### Satisfaction with Life Scale (SWLS; Diener et al., 1985)

This measure has five items presenting statements that respondents are invited to agree or disagree with (on a 7-point Likert-type scale ranging from 1 = “*strongly disagree”* to “*strongly agree”*). Example items include “In most ways my life is close to my ideal” and “I am satisfied with my life.” Scores on each item are averaged to produce an overall score, with higher scores indicating higher life satisfaction. It shows convergent validity with other measures of subjective well-being across two samples (Diener et al., 1985), such as other measures of life satisfaction (*r* = .62 to .66), single-item measures of life satisfaction as a whole (*r* = .62 to .68), semantic-differential measurements of present life (*r* = .59 to.75), and affect balance including relationships with positive affect (*r* = .50 to .51) and negative affect (*r* = −.32 to −.37). Additionally, in their review of the literature, Diener et al. (2013) reported a relationship between satisfaction with life scales, including the SWLS, and non-self-report measures of life satisfaction, such as ratings made by trained raters based on interviews conducted with the participants (Diener et al., 2013). Reliability estimates in past studies have been reported to be.80 or higher (Diener et al., 2013). For our sample, α = .87.

##### Social Connectedness Scale – Revised 15 Item Version (SC-15; Lee et al., 2008)

This scale measures individuals’ sense of belonging to and being in connection with their social world. It was shortened from the 20-item Social Connectedness Scale-Revised (SCS; Lee et al., 2001) to remove items cross-loading across social connectedness and extraversion. Respondents are asked to rate their agreement to statements such as “I am able to relate to my peers” and “Even around people I know, I don’t feel that I really belong” (reverse scored) on a scale from 1 (“strongly disagree”) to 6 (“strongly agree”). Both versions of the scale have convergent validity and are significantly correlated with—but conceptually distinct from—extraversion; *r* = .62 (*p* < .01) for the 20-item version and *r* = .55 (*p* < .01) for the 15-item version (Lee et al., 2008). Additionally, Lee et al. (2008) found the SC-15 to be significantly correlated with life satisfaction (*r* = .50, *p* < .01), affect balance (*r* = .52, *p* < .01), positive affect (*r* = .40, *p* < .01), and negative affect (*r* = −.41, *p* < .01). They also reported good reliability in a general college sample (α = .93). For our sample, α = .94.

##### Meaning in Life Measure (MILM; Hill et al., 2018)

This 8-item measure has two subscales, assessing agreement on a 1 (“*extremely disagree*”) to 9 (“*extremely agree*”) scale with items related to Experience of meaning in life (e.g., “I have something I want to accomplish in my life”) and Reflectivity about meaning in life (e.g., “There are times in my life when I think about what it all means”). Hill et al. (2018) found the measure to have good construct and concurrent validity, showing positive relationships with other measures of meaning in life (*r* = .69, *p* < .001), subjective well-being (*r* = .56-.67, *p* < .001), extraversion (*r* = .26, *p* < .001), agreeableness (*r* = .42, *p* < .001), conscientiousness (*r* = .35, *p* < .001), and openness (*r* = .36, *p* < .001). Hill and colleagues also reported high test-retest reliability, and good internal consistency across the total scale (α = .85) and each of the subscales (α = .82 for Experience and α = .86 for Reflectivity). For our sample, α = .87 for the full scale, α = .79 for the Experience subscale, and α = .88 for the Reflectivity subscale.

##### Meaning in Life Questionnaire (MLQ-Search; Steger et al., 2006)

This measure contains 10 items assessing perceived meaning in life across two subscales: Presence and Search. For this study, we used the five-item Search subscale, which asks participants to rate a series of statements (e.g., “I am always looking for something that makes my life feel meaningful”) on a scale of 1 (absolutely untrue) to 10 (absolutely true). The “Presence” subscale overlaps with the “Experience” subscale in the MILM, and therefore was not included in the survey. Steger and colleagues reported good convergent validity for the Search subscale, showing significant correlations with fear (*r* = .25, *p* < .005), shame (*r* = .19, *p* < .05), sadness (*r* = .26, *p* < .005), neuroticism (*r* = .20, *p* < .05), and depression (*r* = .36, *p* < .005). They also reported good test-retest reliability and high internal consistency of the Search subscale (α = .87) in their undergraduate student samples. For our sample, α = .94.

#### Harms of Social Media Use

##### One-Item Social Media Stress

We developed a stand-alone item asking students to rate their agreement to the statement “social media brings additional stress to my life.” Students indicated their response on a six-point Likert-type scale (1 = “strongly disagree” to 6 = “strongly agree”).

##### Emotional Thermometers (Mitchell, 2007; Mitchell et al., 2010)

This measure asked participants to indicate their level of depression, distress, anxiety, and anger on visual thermometers with “temperature” levels ranging from 0 to 10, where 0 = “none” and 10 = “extreme.” Participants marked the number that best described how much emotional upset they had been experiencing in the week prior to taking the survey, including the day they completed the survey. The thermometers have been validated against other diagnostic measures in a clinical sample and found to have adequate specificity and sensitivity. Specifically, Mitchell (2007) reported in a meta-analysis the pooled sensitivity (77%) and specificity (66%) of the Distress thermometer against various validated measures in ten different studies. Additionally, Mitchell and colleageus (Mitchell et al., 2010) reported that as compared to the Hospital Anxiety and Depression Scale (HADS) total score (Zigmond & Snaith, 1983), the Anger thermometer was optimal with 61% sensitivity and 92% specificity. As compared to the HADS anxiety scale, the Anxiety thermometer was optimal with 92% sensitivity and 61% specificity. As compared to the HADS depression scale (60% sensitivity and 78% specificity) and the DSM-IV screening for major depression (80% sensitivity and 79% specificity), the Depression thermometer was optimal.

##### Three Item Loneliness Scale (Hughes et al., 2004)

This measure was developed as a shortened form of the Revised UCLA Loneliness Scale (R-UCLA; Russell et al., 1980) suitable for inclusion in longer surveys. The three items assess how often respondents lack companionship, feel left out, and feel isolated, measured on a 3-point Likert-type scale (1 = “hardly ever” to 3 = “often”). The average score across the three items is calculated, with higher scores indicating greater loneliness. Hughes et al. (2004) reported that the scale had acceptable internal reliability (α = .72) in older adult samples and was highly correlated with scores on the original R-UCLA scale (r = .82, p < .001) and with scores on loneliness-related items in the Center for Epidemiologic Studies–Depression Scale (CES-D; Turvey et al., 1999), demonstrating convergent validity. For our sample, α = .82.

##### Problematic Social Media Use

This measure is an adaptation of the short-form of the Problematic Internet Use Questionnaire (PIUQ-9; Koronczai et al., 2011), which was developed from the longer 18-item PIUQ (Demetrovics et al., 2008). For the purposes of our study, we changed PIUQ items referring to “the Internet” to refer to “social media.” Respondents were asked to indicate on a scale from 1 (“never”) to 5 (“always”) how frequently they engaged in problematic behaviors on three subscales: Obsession (e.g., “How often do you feel tense, irritated, or stressed if you cannot be on social media for as long as you want to?”), Neglect (e.g., “How often do you neglect household chores to spend more time on social media?”) or Lack of Control (e.g., “How often does it happen to you that you wish to decrease the amount of time spent on social media but you do not succeed?”).

In initially developing the scale through testing with an online community sample of young adults, Demetrovics et al. (2008) reported acceptable/good internal consistency across the three subscales (α ranging from .74 to .87), and high test-retest reliability (r = .903, p < .001). For the briefer version, Koronczai et al. (2011) reported good whole-scale reliability in both adolescent (α = .87) and adult (α = .84) samples. Neither scale has been clinically validated for the diagnosis of internet addiction, although Demetrovics et al. (2008) found that higher PIUQ scores were positively associated with other addictive behaviors, such as the use of slots (*F* = .131, *p* = .011) and other gaming machines (*F* = 4.501, *p* = .025). They also provided support for the validity of the PIUQ as a tool for assessing patterns of use by collecting corresponding data on participants’ internet habits and demographic data. For our sample, α = .86.

##### Online Cognition Scale-Social Comfort Subscale (Davis et al., 2004)

The Online Cognition Scale is a 36-item questionnaire designed to measure problematic Internet use. The full OCS has four subscales (Loneliness/depression, Lack of Impulse Control, Social Comfort, and Distraction). We used only the 13-item Social Comfort subscale for this study, replacing the reference to “online” with reference to “on social media.” Higher Social Comfort is regarded in this study as a further indicator of problematic social media use. Respondents rate their agreement on a 1 (“Strongly disagree”) to 7 (“Strongly agree”) scale for items such as “I say or do things on social media that I could never do in person” and “I wish my friends and family knew how people regard me on social media,” from which an average Social Comfort score is calculated. Davis et al. (2004) reported that the Social Comfort subscale showed (a) convergent validity with related measures of rejection sensitivity (*r* = .41, *p* < .001), procrastination (*r* = .23, *p* < .001), loneliness (*r* = .37, *p* < .001), and feelings of competency online (*r* = .62, *p* < .001), and (b) good reliability (α = .87) in their sample of undergraduate students. For our sample, α = .86.

## Results

Our overall aims were to understand the distinct class profiles of social media use among college students based on the purpose of use, and to quantify how these profiles were associated with beneficial and harmful outcomes. We used LPA, which serves as a person-centered statistical tool. LPA was conducted with Mplus version 8.2 using estimation of robust standard errors to account for non-normality. Akaike (AIC) and Bayesian Information Criterion (BIC) were assessed to select the best-fitting class solution. Lower values of AIC and BIC by at least 10 units suggest an empirically significant better fit. In conjunction, entropy values and significance tests of the Lo-Mendell-Rubin adjusted Likelihood Ratio Test (aLRT) were examined to support empirical identification of a best fitting class solution. Higher entropy values indicate a greater distinction of classes within a solution and a significant LRT indicates that an *n* number of classes is significantly better than an *n-1* model. Interpretability of the fit was also based on significant class membership probabilities. Solutions with class membership probabilities less than 5% were not considered.

Based on the 16 items indicating the purposes of social media use, one to five-class solutions were considered. Model fit comparisons, parsimony, and class membership probabilities were all considered in selecting the best-fitting class solution. Table 1 presents the sequential class solutions. The three-class solution was identified as the best-fitting class solution, primarily using the aLRT tests. Both the AIC and BIC sequentially decreased from one to four-class solutions, and increased in the five-class model, but the aLRT test was not significant for the four-class model. This indicated that the four-class solution did not significantly fit the data better than the three-class model, and that a fourth cluster did not necessarily represent a meaningful class beyond a three-class solution. The entropy for the three-class solution was 0.79, indicating that there was a meaningful distinction between the classes. All class membership probabilities were greater than 5%, with the smallest class representing 25.3% of the sample.

**Table 1:** Sequential class solutions for latent profile analysis

Classes	LL	AIC	BIC	aBIC	Entropy	aLRT
1	−60,872	121,871.715	122,252.343	122,048.993		
2	−58,159	116,576.074	117,343.279	116,933.401	0.790	<0.001
3	−56,971	114,330.634	115,484.415	114,868.009	0.789	<0.001
4	−56,341	113,201.566	114,741.923	113,918.990	0.828	0.8061
5	−55,867	112,382.281	114,309.214	113,279.754	0.824	0.7756

*LL* Log Likelihood, *AIC* Akaike Information Criteria, *BIC* Bayesian

Information Criteria, *aBIC* Adjusted Bayesian Information Criteria, *aLRT*

Lo-Mendell-Rubin adjusted Likelihood Ratio Test

We examined the following results to develop appropriate labels for each class: (a) summary statistics across each profile for each of the 16 purposes of the social media use variable, (b) the number of platforms the respondents used, and (c) the frequency of social media use. The three profiles varied in the level of engagement in each of the social media activities. Figure 1 displays the overall mean responses as well as the means by class across the 16 social media use purposes. Table 2 includes the frequencies of checking social media in general, as well as active and passive use, among the full sample and three groups.

**Table 2:** Means and standard deviations of the types of social media use

	Mean*	*SD*
Active Users (*N = 915, 32.4%*)		
Checking social media account/s	8.32	1.34
Actively using social media	6.00	2.40
Passively using social media	8.18	1.64
Passive Users (*N = 715, 25.3%*)		
Checking social media account/s	6.53	1.92
Actively using social media	3.50	2.16
Passively using social media	6.29	2.17
Average Users (*N = 1198, 42.4%*)		
Checking social media account/s	7.44	1.33
Actively using social media	4.94	2.19
Passively using social media	7.14	1.53
Overall Sample (*N = 2828*)		
Checking social media account/s	7.50	1.64
Actively using social media	4.92	2.44
Passively using social media	7.26	1.89

*Items were rated on a scale of 1 (*never*) to 10 (*all the time*)

### Uses of Social Media

Overall, the participants *primarily* (i.e., “*sometimes*” to “*very ofte*n” on a scale of 1 = “*never*” to 5 = “*very often”*) used social media for nine purposes: entertainment, passing time without a particular purpose, browsing, staying connected with friends and family members, sharing good or interesting things with friends, casually looking at what other people are doing or posting about, seeking information, keeping up with trends, and following sports/fitness (see Fig. 1). On average, the participants used three to four social media platforms (μ = 3.73, SD = 1.24). On a scale of 1 (“*never”*) to 10 (“*all the time”*), on average, participants checked their social media accounts between several times a day and once an hour (μ = 7.50, SD = 1.64), actively updated their social media accounts between once a week and several times a week (μ = 4.92, SD = 2.44), and passively used social media between several times a day and once an hour (μ = 7.26, SD = 1.89).

### Types of Users

When looking at the characteristics of the three-class profiles, an “Active User” group emerged, which represented 32.4% of the sample. Students in this group primarily (at least “sometimes”) used social media platforms for all but three (i.e., seeking advice, venting, and getting income) of the 16 purposes and used them at a higher frequency for these reasons, compared to Average and Passive Users (see Fig. 1). On average, students in this group used more than four different social media platforms—the only group to do so. The “Active Users” checked social media more frequently than the other two groups – between once an hour and several times an hour (μ = 8.32, SD = 1.34) and actively engaged with social media about once a day (μ = 6.00, SD = 2.40), while also passively using social media between once an hour and several times an hour (μ = 8.18, SD = 1.64). In sum, Active Users used more social media outlets for more varying purposes and visited these platforms more frequently than Average and Passive Users (see Table 1).

The second group that emerged was the “Passive Users” group. This group represented 25.3% of the study sample, and primarily used social media for five purposes: to be entertained, pass time, browse, stay connected with friends/family, and casually looking at what other people are doing or posting about. It is important to note that Passive Users used social media for these purposes at frequency levels that were much lower than the overall study sample. They used around 3 different social media platforms (μ = 3.08, SD = 1.26). Students in this group checked social media less frequently than the other two groups – between once a day and several times a day (μ = 6.53, SD = 1.92), actively used social media less frequently – between several times a month to once a week (μ = 3.50, SD = 2.16), and passively used social media less frequently – between once a day to several times a day (μ = 6.29, SD = 2.17).

Lastly, an “Average User” group emerged between the Active Users and Passive Users. This group represented 42.4% of the sample, making it the largest group. This group primarily used social media for nine purposes (all purposes, except for networking, meeting new people, expressing ideas, venting, seeking advice, getting income, and help with school). They reported using between three and four social media platforms (μ = 3.79, SD = 1.15). Overall, Average Users’ patterns of use are less frequent than Active Users’ and more frequent than Passive Users’. They checked social media several times a day to once an hour (μ = 7.44, SD = 1.33), actively used social media close to several times a week (μ = 4.94, SD = 2.19), and passively used social media between several times a day to once an hour (μ = 7.14, SD = 1.53).

### Social Media Use Profile Membership and Psychosocial Outcomes

Once we identified the most appropriate number of class profiles, we used categorical regressions to build models to investigate the relationship between class (the independent variable) and each of the “benefits” and “harms” outcome variables, using the Average Users as the reference group. This allows examination of whether the Active and Passive Users generally scored significantly higher or lower in the outcome variables in relation to Average Users. The “benefits” outcome variables were: perceived social media benefits, life satisfaction, and social connectedness, as well as experience, reflection, and search for the meaning of life. The “harms” outcome variables were: perceived social media stress, distress, anxiety, anger, depression, loneliness, problematic social media use, and social comfort on social media. We used RStudio version 1.1.463 to conduct the categorical regression analyses. To avoid the risk of Type I error in interpreting the regression results, we adopted a conservative alpha of 0.01 to determine significance.

Compared to the Average Users, on average, Active Users had significantly higher levels of all beneficial outcomes, except for satisfaction with life, and higher levels of all the harmful outcomes listed, except for distress and loneliness. Relative to the Average Users, on average, Passive Users reported significantly lower levels of beneficial outcomes on two measures (perceived social media benefits and social connectedness), and lower levels of harmful outcomes on three measures (lower levels of social media stress, problematic social media use, social comfort on social media). Tables 3 and 4 show the complete results of the categorical regression models.

**Table 3:** Categorical regression coefficients of “Beneficial” psychosocial outcomes

Variables	Active Users		Passive Users	
	β	*p*	β	*p*
Social Media Benefits	0.27	<0.001	−0.72	<0.001
Satisfaction with Life	0.29	0.30	0.24	0.42
Social Connectedness	1.69	0.004	−2.75	<0.001
Presence of Meaning	0.29	<0.001	−0.11	0.10
Reflection of Meaning	0.34	<0.001	−0.08	0.32
Search for Meaning	0.31	<0.001	−0.16	0.01

Referent group is Average Users

**Table 4:** Categorical regression coefficients of “Harmful” psychosocial outcomes

Variables	Active Users		Passive Users	
	β	*p*	β	*p*
Social Media Stress	0.33	<0.001	−0.29	<0.001
Distress	0.27	0.02	−0.27	0.03
Anxiety	0.66	<0.001	−0.18	0.19
Anger	0.46	<0.001	−0.16	0.16
Depression	0.32	0.006	−0.12	0.32
Loneliness	0.14	0.05	0.16	0.04
Problematic Social Media Use	1.75	<0.001	−3.04	<0.001
Social Comfort on Social Media	2.09	<0.001	−5.82	<0.001

Referent group is Average Users

## Discussion

The current study examined the social media use patterns among emerging adults (18-25) based on their frequency of social media use for different purposes. As with previous studies that found multiple groups of distinct use (e.g., Bányai et al., 2017; Ilakkuvan et al., 2019; Kurek et al., 2017), we found three unique profiles of individuals who used social media for different purposes: Active, Passive, and Average Users. We also found associations between these profiles and beneficial/harmful psychosocial outcomes. Notably, our study appears to be the first to examine differences in perceived benefits and harms associated with social media use profiles among emerging adults. These associations provide important nuances to understanding how individuals with varying purposes and levels of social media use may reap benefits, while also incurring some psychological costs.

One major trend observed in our findings is that regardless of which profile of social media use individuals fit within, it appeared that students who participated in our study experienced a combination of both beneficial and harmful outcomes associated with social media use. There was no one particular profile that experienced only beneficial or only harmful outcomes. As expected, the Active Users reported significantly higher benefits (except for satisfaction with life) and more harmful psychosocial outcomes (except for distress and loneliness) than the Average Users. The results explain the “double-edged sword” effect of social media, in which the benefits reaped by the Active Users came with psychological costs. In line with previous literature (e.g., Bányai et al., 2017), the Active profile resembled an “at-risk” group for experiencing harmful outcomes. However, our findings on the perceived benefits provided additional insight: perhaps the motive for individuals in this group to continue to actively engage in social media may be from the high level of benefits and sense of connectedness they gained from it, although they also suffered and faced stress from social media use.

Whereas most previous studies on social media use patterns found that passive users were usually associated with harmful outcomes (Frost & Rickwood, 2017; Verduyn et al., 2017), we found nuanced context to understand their experiences by exploring both beneficial and harmful outcomes among emerging adults. As with the Active Users, the Passive Users also reported beneficial and harmful outcomes associated with social media use, but this group appeared to experience significantly lower levels of both sets of outcomes than the Average Users. These individuals reported significantly lower social media stress, problematic social media use, and social comfort on social media. Thus, one might speculate that this group resembles a “low-risk” group. However, our findings also suggest that they experienced fewer benefits associated with social media use—such as lower social connectedness. It appears that Passive Users may experience fewer harmful psychological outcomes, but they may be “missing out” on potential benefits that may be helpful in building relationships and networks.

Of note, meaning in life was a potential outcome of social media use in our study. Despite social media’s potential for facilitating a search for meaning in life (Thomas & Briggs, 2016), no studies have directly examined this process. Given the plethora of information available and the different types of social connections that can be made on social media, it is reasonable to anticipate that individuals may develop greater insight into their meaning in life or use social media to search for meaning. In fact, we found that the Active Users scored significantly higher on the presence of, reflection on, and search for meaning when compared to Average Users. No previous research appeared to connect meaning in life with social media use, but we can speculate as to why this pattern of results emerged. In line with the Uses and Gratifications Theory (Ruggiero, 2000), it is possible that Active Users were more likely to frequently seek information or to form social connections that helped with the development of their own meaning in life. Indeed, Active Users reported the most social connectedness among the three groups. Active Users may depend heavily on online relationships within which they formed their insights about their meaning in life in relation to others in the world. Collectively, our results provide evidence of nuanced processes of social media use that may contribute to emerging adults’ meaning in life and well-being.

### Limitations

We acknowledged that there are several limitations to our study. First, our results were based on a college sample from a university in the Mid-Atlantic region and thus we are not able to generalize our findings to college students in other geographical regions and to non-college populations. Second, although we developed new items that reflect purposes of social media use across multiple domains of use (e.g., social network, information seeking, entertainment), participants may have used social media for other purposes that were not captured in the current set of items. Furthermore, future research would need to examine the psychometric validity of these measures and test to see if our findings may be replicated. Third, given the self-report nature of these items (as well as our outcome measures), we may have captured more subjective, rather than objective, experiences of social media use and its relation to harms and benefits. Therefore, responses may have been subject to self-perception and recall bias. Lastly, causal relations between the variables of interest may not be inferred from our cross-sectional research design.

### Implications for Practice

Despite these limitations, we believe our approach and results have important implications for future practice and research. First, the social media use items we developed and the profile types we identified showed potential as a tool for clinicians and other mental health professionals. Because the current study drew data from a single cross-sectional university student sample, future studies can further validate the social media use items with diverse populations so that it can be used as a tool to assess different dimensions of maladaptive social media use. The three profiles may be used to conceptualize people’s patterns of social media use and identify related psychological risks and protective factors. Second, to date, most studies on social media use patterns have examined primarily harmful outcomes. The most salient contribution of our study is that we examined the benefits as well as the costs associated with social media use. This enabled us to identify a “double-edged sword” effect of experiencing both harms and benefits, which has important implications. Our results suggest that while Active Users experienced “costs” from their social media use, they also perceived that they were benefiting from their social media use, which may have motivated them to actively use social media more. It is important for clinicians, educators, and other professionals to understand these perceived benefits, while being wary of the harmful effects of problematic or frequent social media use.

### Implications for Future Research

For future research, we believe it is important to examine the mechanisms for social media harms and benefits, and to test potential interventions to minimize the harms, while maintaining or enhancing the benefits. Additionally, it would be important to conduct research on approaches that can promote the benefits more effectively, especially among Passive Users that seem to be “missing out” on the benefits of social media use, while also helping Active Users to understand the concurring costs of social media. Moreover, future studies should consider predictors of the different patterns of social media use. Although we have identified three descriptive clusters of social media use, we are limited in what we can say about the factors that lead to such patterns. Many factors may differentiate adults’ social media use, including individual differences (e.g., personality differences), group factors (e.g., collective self-esteem, group social identity), and offline social contextual variables such as relationship satisfaction and sense of belongingness. For example, it would be important for future researchers to assess how the benefits and harms fof social media use may be contextualized among underrepresented and marginalized youth and emerging adults (e.g., racial minority individuals, LGBTQ individuals). As Dari et al. (2021) suggested, community-based participatory research could be used to explore ecologically valid and relevant lived experiences of social media use that can inform how the harms may involve experiences such as online racism (Keum & Miller, 2017, 2018) and benefits can include culturally-relevant social support networks (Keum, 2017). Another opportunity for future research would be to examine differences in psychosocial outcomes based on social media platforms. There is evidence from social media marketing research that social media users may engage with social media platforms differently, which could lead to different psychosocial outcomes (Goodrich & de Mooij, 2013). In addition to quantitative methods, researchers can consider innovative qualitative approaches such as the Online Photovoice method which gives participants opportunities to express their own lived experiences online with as little bias and influence from the researchers (Tanhan & Strack, 2020). Finally, our data were collected prior to the start of the COVID-19 pandemic. It is unclear how the reliance on internet connections during the pandemic may have influenced young adults’ social media use and its relationship with psychosocial well-being. The interaction between COVID-19-related psychosocial implications and social media use would need to be studied.

## Conclusion

In conclusion, with the ever-growing presence and influence of social media on the day-to-day lives of emerging adults, who are going through a critical developmental stage, it is crucial that the full extent of the effects on users’ psychological and overall well-being are thoroughly investigated and understood. The knowledge base that our study has added can inform educational and other interventions aiming to optimize the benefits and minimize the harms associated with social media, and help emerging adults navigate the right balance in this digital world.

## Data Availability

The datasets generated during and/or analyzed during the current study are not publicly available due to confidentiality and privacy reasons set forth by the Institutional Review Board.
